# Synthesis, Crystal Structure, Hirshfeld Surface, RDG, ELF, LOL, DFT, and Molecular Docking Studies of a Binuclear Copper(II) Carboxylate Complex

**DOI:** 10.1002/open.70231

**Published:** 2026-05-20

**Authors:** Abiodun Atoyebi Ajibola, Mehran Feizi‐Dehnayebi, Senem Akkoc, Lesław Sieroń, Waldemar Maniukiewicz

**Affiliations:** ^1^ Department of Chemical Sciences Thomas Adewumi University Oko‐Irese Kwara State Nigeria; ^2^ Department of Basic Pharmaceutical Sciences Faculty of Pharmacy Suleyman Demirel University Isparta Türkiye; ^3^ Faculty of Engineering and Natural Sciences Bahcesehir University Istanbul Türkiye; ^4^ Institute of General and Ecological Chemistry Lodz University of Technology Lodz Poland

**Keywords:** copper(II) complex, DFT calculations, Hirshfeld surface analysis, hydrogen‐bonding networks, phenylacetate ligand

## Abstract

This study describes the fabrication and examination of binuclear copper(II) phenylacetate (PAA) complex with metronidazole (mnz), formulated as [Cu_2_(μ‐PAA)_4_(mnz)_2_](mnz)_2_(H_2_O)_2_ (**1**). The complex was synthesized at room temperature and the product was characterized via FTIR, UV–Vis, and PXRD. The single‐crystal X‐ray diffraction of complex **1** reveals that it crystallizes in the triclinic space group P‐1. The SCXRD illustrated that complex **1** adopts a bidentate bridging mode with an almost ideal square‐pyramidal geometry (*τ*_5_ descriptor). Complex **1** is stabilized by extended hydrogen‐bonding networks, as confirmed by Hirshfeld surface analysis and 2D fingerprint plots. Quantitative analysis demonstrated that H···H interactions dominate the crystal packing (49.6% in **1**), indicating that van der Waals forces are the major contributors to solid‐state stability. DFT calculations confirmed the structural stability and electronic features of the binuclear copper complex. FMO analysis revealed a narrow HOMO–LUMO energy gap (1.39 eV), indicating enhanced electronic responsiveness and chemical reactivity. RDG analysis indicated that molecular stability arises from a balance of coordination‐driven attractive interactions, weak dispersive forces, and localized steric effects. Furthermore, docking study against the SARS‐CoV‐2 main protease showed favorable binding affinity (−7.97 kcal/mol), suggesting potential biological relevance of the complex as a metal‐based bioactive scaffold.

## Graphical abstract

A binuclear copper(II) phenylacetate–metronidazole complex was successfully synthesized and structurally characterized. Hirshfeld surface analysis confirmed dominant H···H interactions governing crystal packing stability. DFT calculations demonstrated strong agreement with experimental geometry. RDG, ELF, and LOL analyses indicated stabilization through coordination bonding and weak dispersive interactions. Molecular docking showed favorable binding affinity toward the SARS‐CoV‐2, suggesting potential bioactivity.

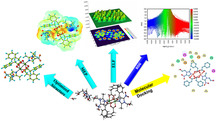

## 1 Introduction

Copper(II) complexes are among the most widely studied first‐row transition metal compounds, due to their diverse structural architectures, catalytic properties, and promising biological applications [1, 2, 3]. In copper carboxylate chemistry, carboxylate ligands are known to adopt multiple coordination modes, giving rise to unique structural frameworks with distinct physicochemical and functional properties [4, 5, 6].

Phenylacetic acid (PAA), a naturally occurring aromatic carboxylate, possesses notable antimicrobial and antifungal activities [7]. It has been shown to form stable complexes with transition metals, particularly copper(II) and zinc(II). Numerous studies have examined the coordination chemistry of phenylacetate with 3d transition metals, demonstrating their structural diversity and a broad range of physicochemical and biological properties. These complexes have been investigated for their antibacterial, antifungal, DNA binding, cytotoxic, and magnetic behaviors, underscoring their significance in coordination chemistry and potential functional applications [8, 9, 10, 11, 12, 13, 14].

Despite these findings, the coordination chemistry of PAA remains relatively underexplored, leaving ample scope for further investigation. A deeper understanding of PAA‐based complexes could provide valuable insights into their structural chemistry and potential pharmaceutical applications. To enhance the therapeutic relevance of copper(II) phenylacetate complexes, the incorporation of auxiliary ligand such as metronidazole represents a promising approach. This ligand typically acts as monodentate donor and is well recognized for its medicinal importance [15, 16, 17, 18, 19].

Metronidazole (mnz), [1‐(2‐hydroxyethyl)‐2‐methyl‐5‐nitroimidazole], a clinically established antimicrobial and antiparasitic drug, has previously been utilized as a coligand in transition metal complexes, to enhance pharmacological activity [15, 17]. Metronidazole is a redox‐active antimicrobial prodrug with significant immunomodulatory effects, capable of suppressing proinflammatory cytokines and reducing oxidative stress, particularly in COVID‐19‐associated inflammation [20]. It also interacts directly with key cytokines, especially IL‐12, inhibiting cytokine–receptor binding through structural modulation. This supports its potential as a repurposable therapeutic for COVID‐19 and other inflammatory disorders [21]. This study describes the fabrication, characterization, and structural analysis of a copper(II) phenylacetate complex using metronidazole as a coligand. The complex was analyzed through single‐crystal X‐ray diffraction and complementary spectroscopic methods. Furthermore, DFT insights were used to study the electronic features and stability of the compound, while molecular docking simulation was explored to study the possible interactions with biological target, including SARS‐CoV‐2 main protease.

## 2 Experimental

### 2.1 Materials and Methods

All solvents and reagents used in the synthesis were of analytical grade and utilized directly without further purification. The melting point of compound **1** was determined using a BMP‐1C Digital Melting Point Apparatus.

### 2.2 Synthesis

#### 2.2.1 Synthesis of [Cu_2_(μ‐PAA)_4_(mnz)_2_](mnz)_2_(H_2_O)_2_ (1)

Phenylacetic acid (2 mmol, 0.272 g) and NaHCO_3_ (2 mmol, 0.168 g) were added to ethanol/H_2_O (1:1, 20 mL) and stirred for 30 min. Subsequently, Cu(NO_3_)_2_ · 3H_2_O (2 mmol, 0.483 g) was introduced, producing a blue solution. Metronidazole (2 mmol, 0.342 g) was then added, causing the solution to turn green, and the mixture was stirred for 3 h. A green precipitate was filtered out and air dried. Slow evaporation of the green solution over 4 days afforded green quality single crystals (**1**) which were filtered and air dried. Yield: 52.73%.

### 2.3 Spectra Characterization

The UV–Visible spectrum of complex **1** was measured with a UV‐6300 PC spectrophotometer, employing DMSO as the solvent. The FTIR spectrum of complex **1** was obtained in the 4000–400 cm^−1^ region using KBr pellets on a SHIMADZU FTIR‐8400S spectrometer (Shimadzu Scientific Instruments).

### 2.4 Crystal Structure

Single‐crystal X‐ray diffraction was performed at 100 K on a RIGAKU XtaLAB Synergy diffractometer employing MoKα radiation (*λ* = 0.71073 Å). The raw data were processed, including integration, scaling, reduction, and absorption correction, using CrysAlis PRO software [22]. The crystal structure was determined through direct methods with SHELXT 2018/3 [23] and refined by full‐matrix least‐squares analysis on *F* ^2^ using SHELXL 2018/3 [24]. All nonhydrogen atoms were refined anisotropically. Hydrogen atoms participating in hydrogen bonding were identified from Fourier difference maps and refined without constraints, whereas those bound to carbon were introduced at geometrically estimated positions (C–H = 0.93–0.98 Å) and refined with a riding model, with isotropic displacement factors set to 1.2–1.5 times the Ueq of the parent carbon. Molecular illustrations were prepared using Mercury software [25]. The recent crystallographic methodology based on PLATON or CSD statistics lists typical geometric cut‐offs used for interaction analysis, including H···Cg < ∼3.6 Å and X–H···Cg > 90° [26]. These values arise from accepted small‐molecule crystallography standards and statistical distributions of close contacts. The level C alerts PLAT906 and PLAT911 in a CIF check report are generally not critical. Both alerts are relatively common and usually acceptable. PLAT906—Large K value in the analysis of variance. This alert suggests that the weighting scheme used during refinement may not be optimal. PLAT911—Missing reflections in the final refinement. This alert indicates that some measured reflections (within the expected resolution range) were omitted from the refinement. This may be due to filtering of outliers and experimental limitations. Key crystallographic data and refinement data are presented in Table 1.

**TABLE 1:** Crystal parameters for complex **1**.

Parameters	1
Empirical formula	C_44_H_46_Cu_2_N_6_O_14_,2(C_6_H_9_N_3_O_3_),2(H_2_O)
Formula weight	1388.32
Temperature, K	100
Crystal system	Triclinic
Space group	P‐1
*a*, Å	8.7651(1)
*b*, Å	10.4015(1)
*c*, Å	18.7933(2)
*α*, °	79.908(1)
*β*, °	87.468(1)
*γ*, °	67.886(1)
Volume, Å^3^	1562.34(3)
Z	1
Density, Mg/m^3^	1.476
Absorp. coeff., mm^−1^	1.586
F(000)	722
Crystal size, mm^3^	0.07 × 0.15 × 0.25
Radiation	CuK_α_
Theta range for data collection, °	4.7, 67.7
Index ranges	−10: 10; −11: 12; −22: 22
Reflection collected	56 898
Independent reflections	5655, [*R*_int_ = 0.024]
Goodness of fit on *F* ^2^	1.18
Final *R* indices [*I* > 2*σ*(*I*)]	0.0335, [wR_2_ = 0.0764]
Largest diff. peak and hole, eÅ^−3^	0.48, −0.40

### 2.5 Powder X‐Ray Diffraction (PXRD)

PXRD measurements were carried out on a PANalytical X’PERT PRO diffractometer equipped with Cu Kα radiation (*λ* = 1.54056 Å), operated at 40 kV and 30 mA, utilizing a Ni filter and an X’celerator detector. Data were collected over a 2*θ* range of 4°–80°, with a step interval of 0.017° and a counting time of 30 s per step. Comparison between the calculated and experimental patterns verified the phase purity of the synthesized microcrystalline samples (Figure S1).

### 2.6 Hirshfeld Surface (HS) Investigation

HS study was performed to examine the characteristics of intermolecular interactions within the crystal structures. These surfaces were constructed using electron densities derived from the sum of spherical atomic contributions. For each crystal system, the HS is uniquely defined once the set of spherical atomic electron densities is specified, enabling a detailed examination of intermolecular contacts across the lattice. At every point on the surface, two parameters are considered: *d*_e_, the distance from the surface point to the closest nucleus outside the surface, and *d*_*i*_, the distance to the nearest nucleus inside the surface [27]. In this work, the normalized distance values ranged from −0.7273 to 1.3936 for compound **1**. These parameters, visualized through 2D fingerprint plots [28], provide a quantitative representation of the intermolecular interactions involved. The HSs in this study were mapped over *d*_norm_, and the corresponding fingerprint plots were generated using CrystalExplorer version 21.5 [29].

### 2.7 DFT Investigation

The molecular geometry of the synthesized complex, **1**, was optimized using DFT at the B3LYP level. The 6‐311g (d,p) basis set was employed for nonmetal atoms (C, H, N, and O), while the LANL2DZ ECP/basis set was used for Cu to account efficiently for core relativistic effects with established performance for transition‐metal complexes. The calculation was conducted using Gaussian 09W [30] with molecular visualizations carried out via GaussView 6.0. Geometry optimizations and subsequent frequency analyses, performed in the gas phase, confirmed that the structure corresponds to true minimum [31], as no imaginary frequencies were detected. This optimized structure served as the foundation for further computational analyses, including molecular electrostatic potential (MEP) surfaces, FMO, and key quantum chemical descriptors. This unified computational approach allowed for a comprehensive evaluation of molecular reactivity, stability, and site‐specific chemical behavior.

### 2.8 RDG, ELF, and LOL Analysis

RDG analysis, RDG scatter plot, electron localization function (ELF), and localized orbital locator (LOL) calculations were performed using the Multiwfn program based on the wavefunctions obtained from optimized geometries. RDG analysis was employed to identify noncovalent interactions through sign (*λ*_2_)*ρ* versus RDG plots and corresponding isosurfaces, while ELF and LOL analyses were used to investigate electron localization and bonding characteristics [32, 33]. The resulting isosurfaces were visualized using graphical visualization software.

### 2.9 Docking Simulation

Molecular docking is widely used as a computational strategy to investigate receptor + ligand interactions and to estimate their associated binding affinities and interaction energies [34]. The target protein selected for this study was the SARS‐CoV‐2 main protease (Mpro), as it plays a crucial role in viral replication and transcription, making it one of the most validated drug targets for anti‐COVID‐19 drug discovery. The crystal structure of Mpro complexed with the N3 inhibitor (PDB ID: 7BQY) was chosen due to its high structural resolution (1.70 Å), which ensures accurate representation of the active site geometry and reliable docking results. Molecular docking was done to investigate the anti‐COVID‐19 effects of compound **1**, employing AutoDock 4.2 [35] together with AutoDock Tools 1.5.6. The crystal structure of the SARS‐CoV‐2 main protease bound to the N3 inhibitor (PDB ID: 7BQY) was retrieved from the RCSB (www.rcsb.org). Prior to docking, the structure was refined by removing water molecules, cocrystallized ligands, and other nonessential atoms. Polar hydrogen atoms were introduced, and Kollman charges were assigned to improve the precision of the docking process. The molecular geometry of **1** was optimized using DFT prior to docking. The binding site was defined by constructing a grid box over the protease's active site, with a spacing of 0.375 Å and dimensions of 100 × 100 × 100, centered on the coordinates of the 7BQY structure. Docking was performed using the LGA with standard settings [36, 37]. The resulting binding conformations were visualized and analyzed utilizing Discovery Studio Visualizer 4.1 for interaction profiling and structural insight.

## 3 Results and Discussion

### 3.1 Crystal Structure

**FIGURE 1:**
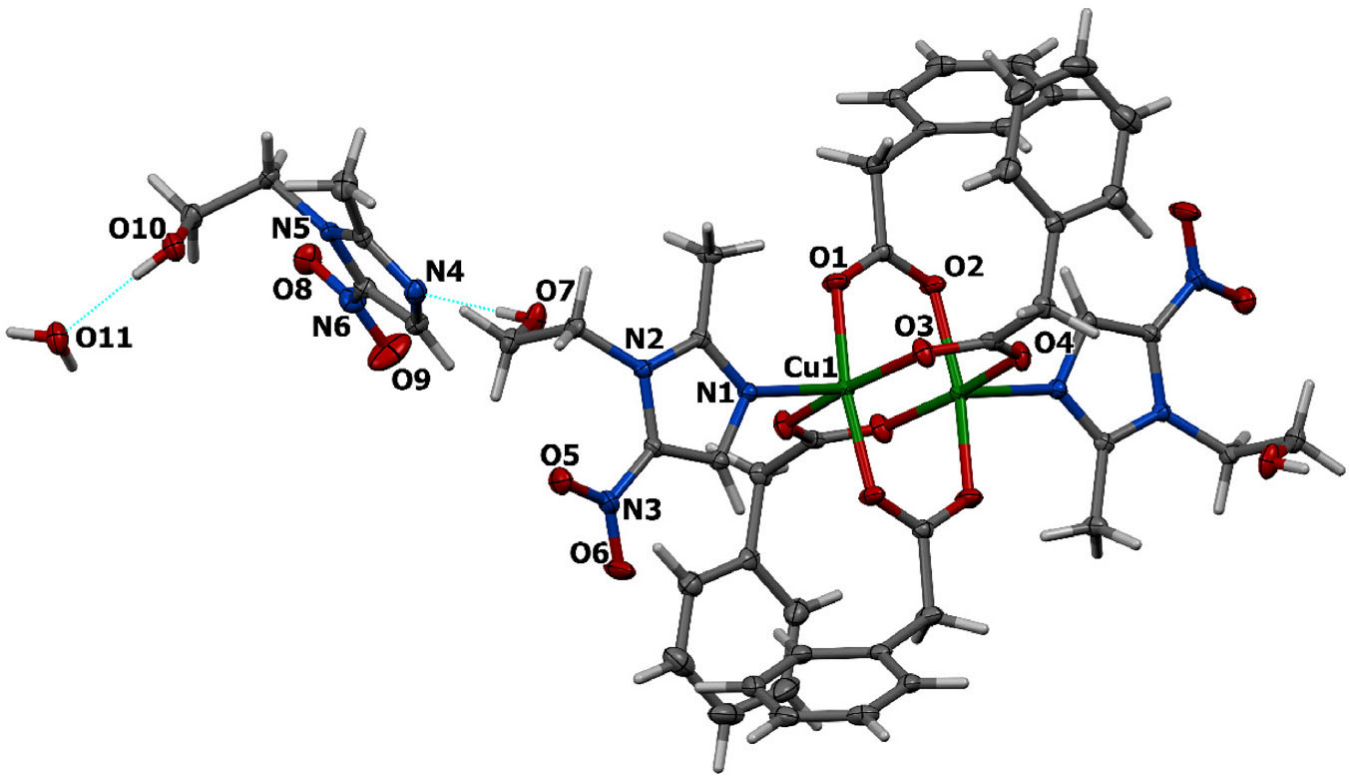
Structural diagram of **1**, including the atom numbering system. Atoms other than hydrogen are represented by displacement ellipsoids with a 50% probability level.

**FIGURE 2:**
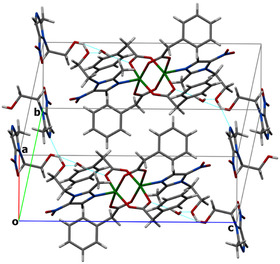
Packing diagram of **1** along the *c*‐axis. Hydrogen bonds are indicated with dashed lines.

The single‐crystal X‐ray diffraction reveals that complex **1** crystallizes in the triclinic space group P‐1. An ORTEP representation of the compound and the view of its packing pattern are demonstrated in Figures 1 and 2, respectively. The selected parameters are presented in Table S1. The molecular unit of compound **1** comprises centrosymmetric binuclear copper centers with four bidentate, metal‐bridging phenylacetic acid ligands and two monodentate metronidazole acting as terminal ligands. Additionally, another metronidazole and water molecules are present in the molecular lattice. In the binuclear structure, the four phenylacetic acid bridging moieties exhibit a typical paddle wheel arrangement about the Cu–Cu axis, similar to those observed in many binuclear copper–carboxylate complexes [19]. The copper centers adopt an almost ideal square‐pyramidal geometry, as indicated by the value of *t* descriptor for 5‐coordination [*τ* = 0.002 (*τ *= |*α*‐*β*|/60)] [38] where *α* and *β* are the largest basal angles around the metal center. The basal plane around each Cu(II) center is formed by four oxygen atoms (O1, O2, O3, and O4) of the phenylacetic acid ligands, while the N1 nitrogen donor atoms from metronidazole ligand occupies the apical position for Cu1 atom. The Cu‐O bond lengths in the basal plane range from 1.9615(16) to 1.9853(17) Å, while the Cu1–N1 apical bond length is observed to be 2.1609(18) Å. The copper atom is displaced from the basal plane toward N1 atom by 0.2121(3) Å. Additionally, a short Cu⋅⋅⋅Cu interaction (2.658 Å) is present, contributing to the stabilization of the paddle‐wheel binuclear copper(II) unit.

The hydrogen‐bonding parameters for complex **1** is summarized in Table S2. Complex **1** exhibits an extended hydrogen‐bonding network, which plays a crucial role in stabilizing their supramolecular architectures. In complex **1**, the hydrogen‐bonding pattern is dominated by classical O–H⋅⋅⋅O and O–H⋅⋅⋅N interactions. A strong intramolecular O7–H7A⋅⋅⋅N4 hydrogen bond indicates a nearly linear geometry and contributes significantly to the structural stabilization. Additional hydrogen bonds, such as O10–H10⋅⋅⋅O11 and O11–H11A⋅⋅⋅O7^#1^ (symmetry code #1: 1−*x*, 1*−y*, *−z*), form intermolecular linkages that generate a 3D supramolecular framework. Several weak C–H⋅⋅⋅O interactions further reinforce the packing arrangement, highlighting the cooperative role of weak contacts in consolidating the crystal structure.

Furthermore, the crystal structure of **1** reveals distinct patterns of *π*···*π* stacking contacts and C–H···*π* or O–H···*π* contacts (Tables S3 and S4) that significantly influence their supramolecular organization. In complex **1**, two well‐defined *π*···*π* stacking interactions are observed. The first involves the overlap of a five‐membered N1–C19 ring with an adjacent six‐membered C11–C16 ring, displaying a centroid–centroid separation of 3.659 Å and a very small interplanar tilt (*α *≈ 5°), indicative of a typical parallel‐displaced geometry. The second interaction is formed between two symmetry‐related five‐membered rings (N4–C25), with a centroid distance of 3.708 Å and an almost perfect parallel orientation. These *π*‐stacks are complemented by additional C–H···*π* and O–H···*π* contacts. Notably, a C20–H20C···*π* interaction (H···Cg = 2.68 Å) provides extra stabilization by linking alkyl substituents to aromatic surfaces, thereby reinforcing the packing arrangement.

### 3.2 HS Analysis

**FIGURE 3:**
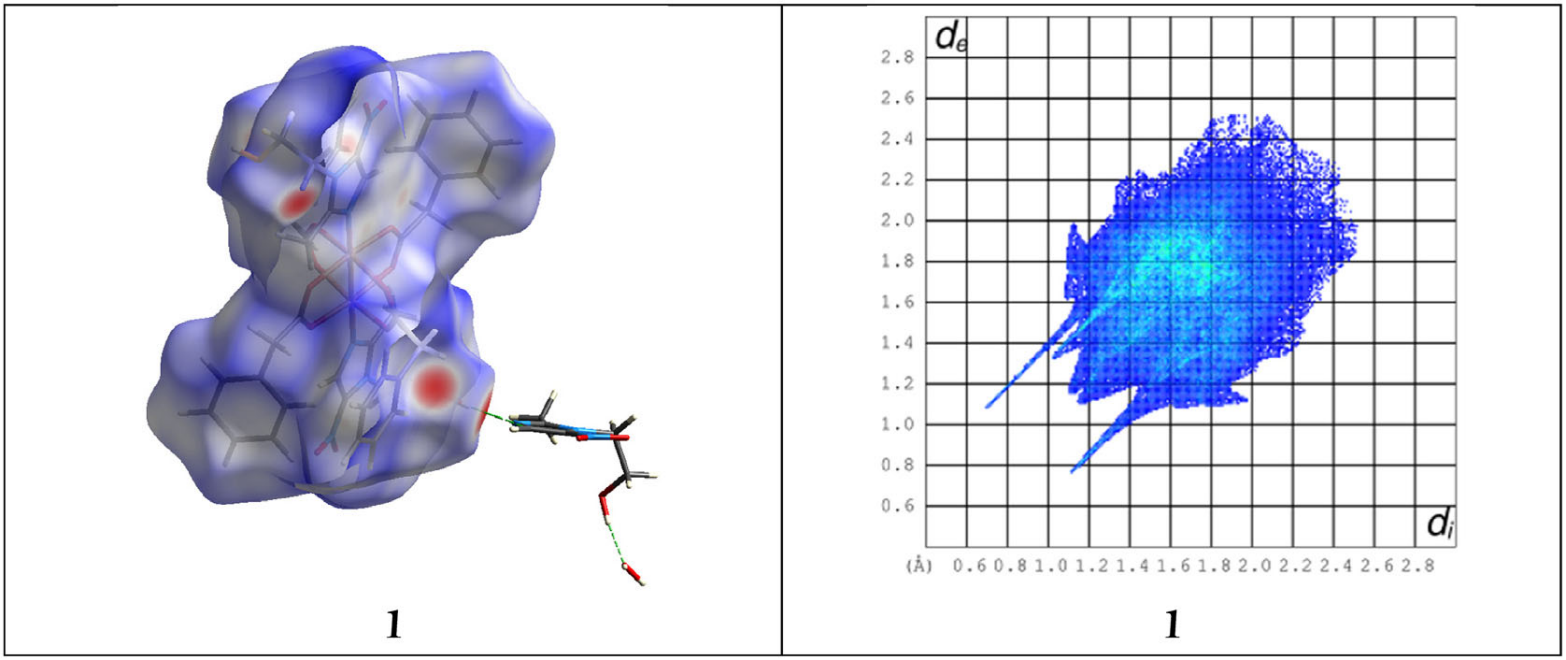
HS of **1** (left) visualized using *d*_norm_, alongside the related 2D fingerprint maps (right) depicting all intermolecular interactions.

Following the structural pattern observed in the solid‐state forms of analyzed compounds, we aimed to quantify the noncovalent interactions that contribute to their supramolecular architecture. In this study, the intermolecular contacts responsible for the formation of supramolecular assemblies were evaluated. HS analyses of the compounds were performed and mapped over *d*_norm_ (Figure 3, left column) for complex **1**. The corresponding fingerprint plots (Figure 3, right column) display scattered points that clearly highlight the various intermolecular contacts present within the crystal structures. In the HS, negative *d*_norm_ values are represented in red, indicating contacts that are shorter than the combined van der Waals (vdW) radii of the interacting atoms. Regions shown in white correspond to intermolecular distances that are approximately equal to the sum of the vdW radii, where *d*_norm_ is zero. Conversely, contacts longer than the sum of the vdW radii, reflected by positive *d*_norm_ values, are displayed in blue.

**FIGURE 4:**
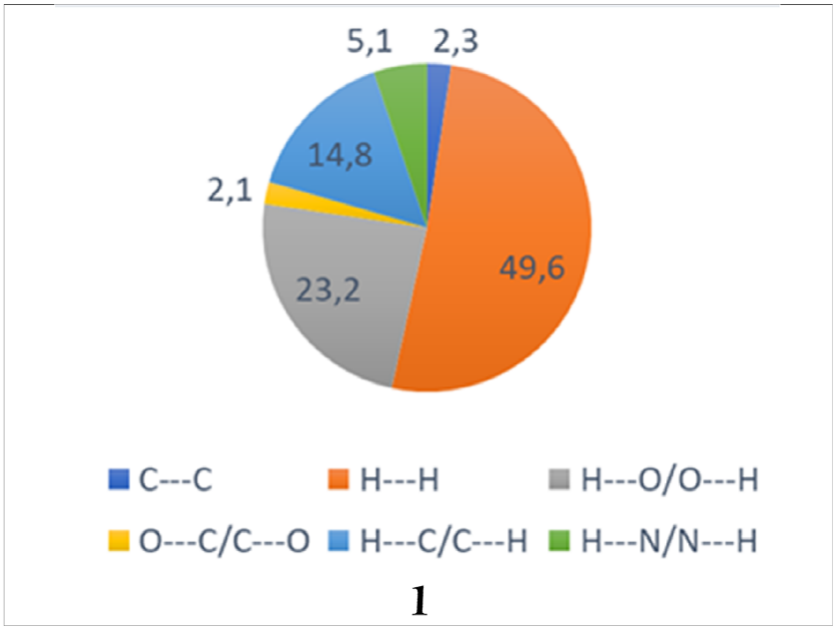
Summary of the various contact contributions (>2.0%) in Hirshfeld surface area for the analyzed complex.

The HS analyses of **1** enable a quantitative assessment of the intermolecular contacts. The decomposed 2D fingerprint plots provide the relative contributions of different types of contacts (Figures 4 and S2). For **1**, H···H interactions dominate the crystal packing, accounting for ca. 49.6% of the total surface. This result indicates that vdW forces are the most significant contributors to the stabilization of the solid‐state structures. In **1**, a substantial contribution arises from H···O/O···H interactions (23.2%), reflecting the importance of O–H···O and C–H···O hydrogen bonds in linking molecular units. The H···C/C···H contacts (typically associated with C–H···*π* interactions) contribute 14.8% in **1** structure. Furthermore, C···C contacts associated with *π·*··*π* stacking interactions contribute only 2.3% of the total intermolecular contacts, indicating that these interactions play a minor role in the crystal packing of complex **1**. When compared to the related binuclear copper carboxylate complexes [19] (complex 1‐SA: 45.5% H···H, 30.0% O···H; complex 2‐PPA: 45.1% H···H, 26.3% O···H), these values appear to be typical and consistent for this class of compounds. In all three structures, H···H contacts are the most significant contributors, ranging from approximately 45% to 50%. This is typical for paddle‐wheel binuclear copper carboxylates featuring bulky organic ligands (such as phenylacetate, sorbate, or 3‐phenylpropanoate) and N‐donor ligands like metronidazole. The high percentage reflects the abundance of aliphatic and aromatic CH groups on the periphery of the molecules, which engage in extensive vdW contacts that stabilize the crystal lattice. The O···H/H···O interactions in the manuscript (23.2%) are slightly lower than those in the sorbic acid complex (30.0%) but comparable to the 3‐phenylpropanoic acid complex (26.3%). The H···C/C···H contributions in **1** align closely with those in the comparative article (17.4% and 18.1%). These values are characteristic of structures with multiple aromatic rings, where T‐shaped C–H···*π* interactions provide additional supramolecular stability.

### 3.3 FTIR Spectrum of **1**

**FIGURE 5:**
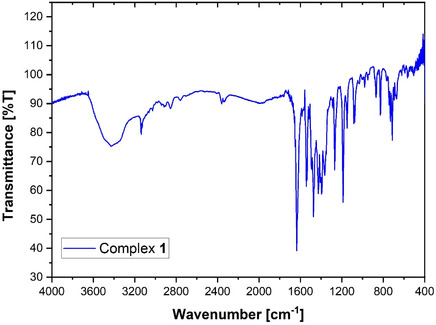
FTIR spectrum of complex **1**.

FTIR spectrum of the title complex was recorded over the range 4000–400 cm^−1^, as shown in Figure 5. Complex **1** reveals a broad absorption band in the range 3500–3200 cm^−1^, attributable to O–H stretching vibrations of water molecules, consistent with the presence of lattice and/or coordinated water. The most significant features in the IR spectrum of **1** are the asymmetric and symmetric stretching vibrations of the carboxylate groups of the phenylacetate ligand.

For complex **1**, the *ν*_as_(COO^−^) and *ν*_s_(COO^−^) bands appear at 1636 and 1474 cm^−1^, respectively. The corresponding separation, Δ*ν *= *ν*_as_ − *ν*_s_, is 162 cm^−1^. Since this Δ*ν* value is below 200 cm^−1^, it confirms the bidentate bridging coordination mode of the phenylacetate carboxylate moiety, in agreement with established crystallographic data. Additionally, complex **1** shows a band at 1535 cm^−1^ assigned to the *ν*(C=N) stretching vibration of the noncoordinated metronidazole molecule. In contrast, the coordinated metronidazole exhibits a *ν*(C=N) band shifted to approximately 1543 cm^−1^, indicating involvement of the imine nitrogen in metal coordination. These vibrational features are fully consistent with the coordination environment observed in the single‐crystal X‐ray structure.

### 3.4 UV–Vis Spectrum of **1**

**FIGURE 6:**
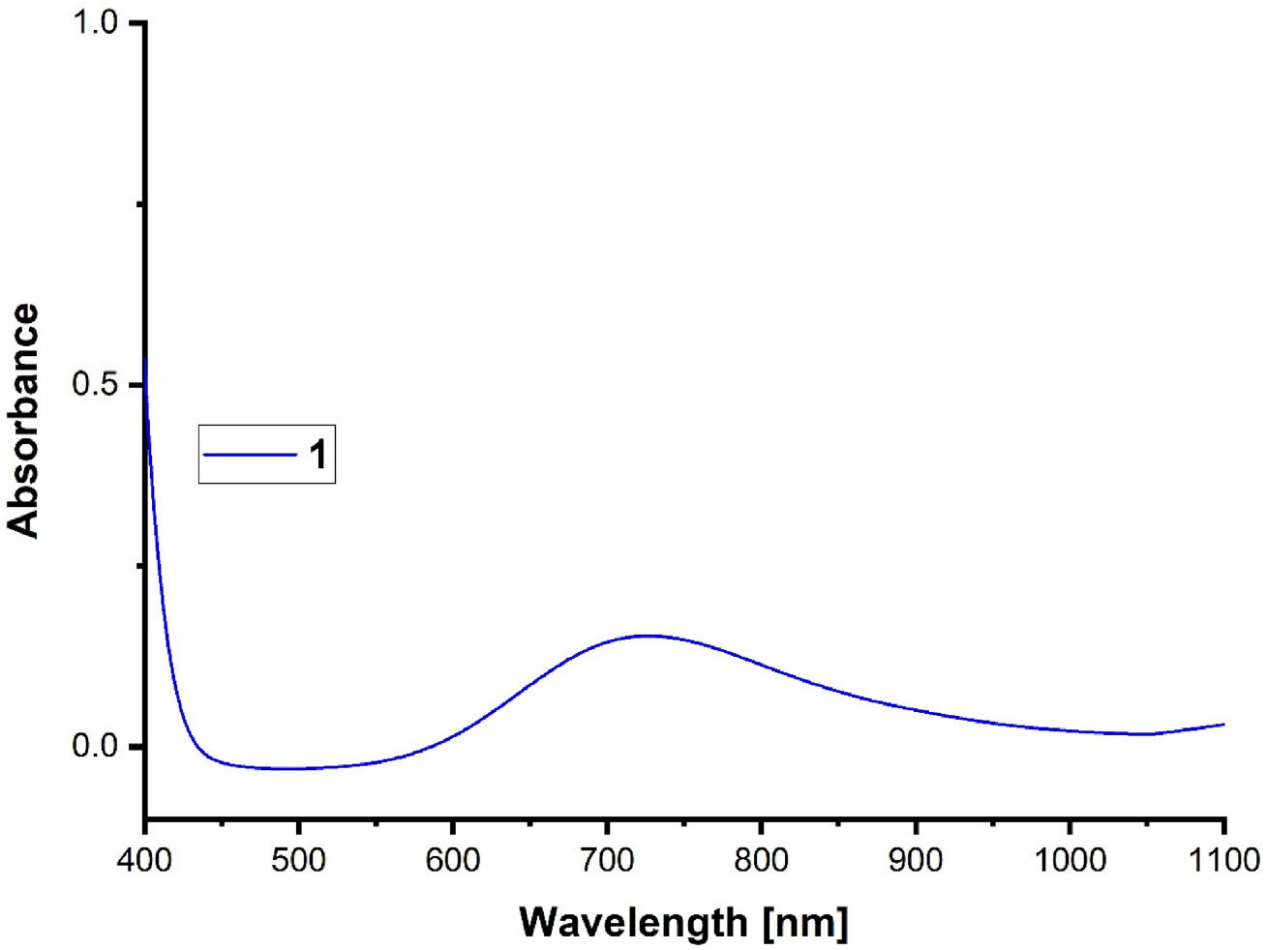
UV–Vis spectrum of complex **1**.

The UV–Vis spectrum of complex **1** was measured at ambient temperature from 400 to 1100 nm in DMSO (Figure 6). The electronic spectrum of the dinuclear Cu(II) complex exhibited a broad absorption band centered at approximately 723 nm, which is assigned to d–d transitions consistent with a square‐pyramidal coordination environment around each Cu(II) center. In the complex, each Cu(II) ion is equatorially bridged by four phenylacetate ligands and axially coordinated by a nitrogen donor atom from metronidazole.

### 3.5 Computational Studies

#### 3.5.1 DFT Calculation

**FIGURE 7:**
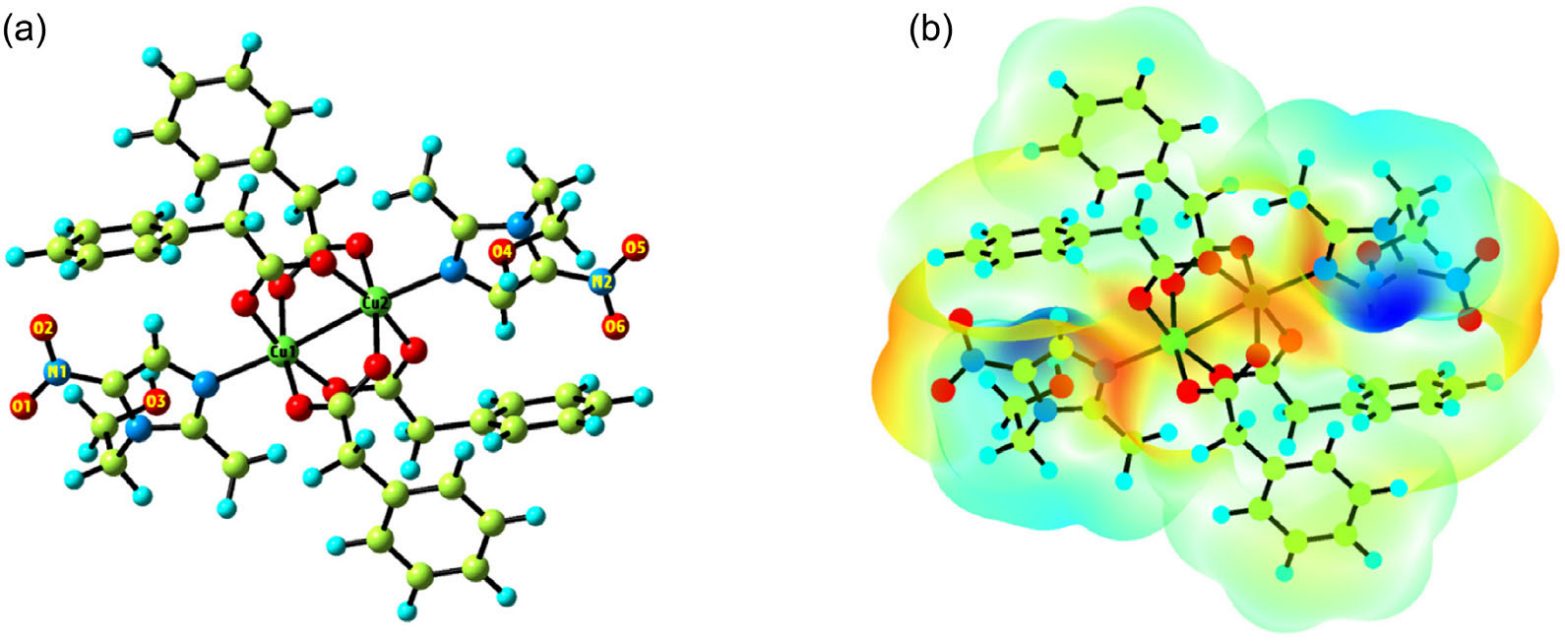
Optimized structure (a) and MEP illustration (b) of complex **1** under DFT method.

The molecular geometry of the copper complex **1** was optimized via DFT calculation. The calculation employed the B3LYP exchange‐correlation functional under gas‐phase conditions. To ensure accuracy across different atomic species, a dual‐basis approach was implemented: the 6‐311g (d,p) basis set was utilized to all nonmetal atoms, while the LANL2DZ effective core potential was assigned to the copper centers. Figure 7a depicts the optimized geometry, including key atom labels for clarity. In complex **1**, the Cu1 center forms coordination bonds with one nitrogen atom, its counterpart Cu2, and four oxygen atoms. Similarly, Cu2 coordinates to one nitrogen atom, Cu1, and four oxygen atoms, reflecting a symmetric metal‐ligand environment that stabilizes the bimetallic core. This configuration underscores the flexibility and diverse binding modes copper can adopt in different ligand environments. The computed ground‐state energy for **1** is found to be −3479.13 Hartree. This negative value reflects the thermodynamic favorability of complex **1** demonstrating great stabilization which is likely due to its extended delocalization and metal–metal interactions within the binuclear framework. Furthermore, the calculated bonding parameters, derived from DFT, exhibit excellent consistent with the experimental values obtained from X‐ray crystallography, confirming the reliability of the computational models for complex **1**.

#### 3.5.2 MEP Illustration

The MEP map is an effective tool for identifying regions susceptible to electrophilic and nucleophilic attack, while also providing insight into potential hydrogen bonding interactions [39, 40, 41]. The MEP map for complex **1** was derived from the Gaussian computational output and is presented in Figure 7b. In the complex **1**, the blue‐shaded regions surrounding the hydrogen atoms bonded to oxygen atoms O3 and O4 underscore their pronounced electrophilic nature. This strong electron deficiency makes them particularly reactive toward nucleophilic species. In contrast, the intense red coloration concentrated around oxygen atoms O1, O2, O5, and O6, as well as in the central region of the molecule, indicates zones of higher electron density. These areas, rich in electronegative elements such as oxygen and nitrogen, are likely targets for electrophilic interaction due to their nucleophilic character.

#### 3.5.3 Frontier Molecular Orbitals (FMOs)

**FIGURE 8:**
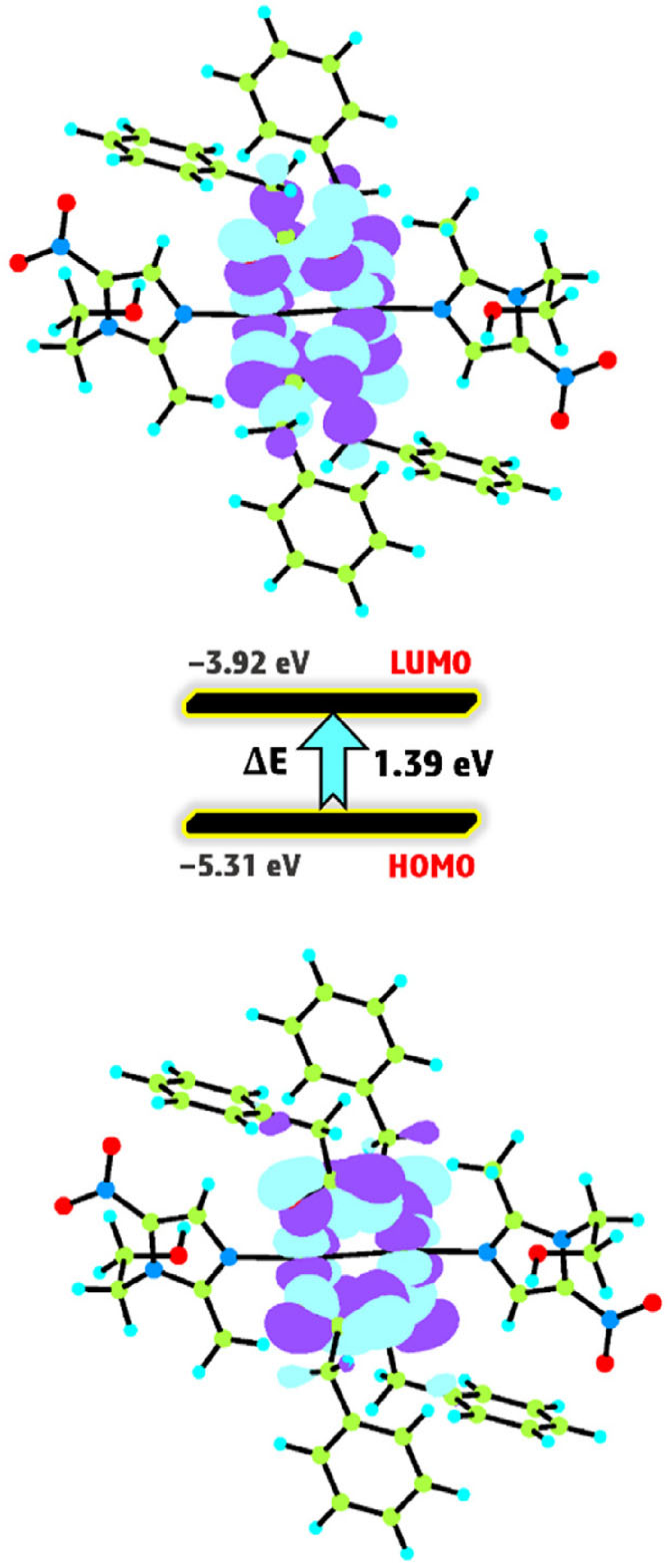
The FMO map of **1** at B3LYP level.

The FMO, namely HOMO and LUMO, serve as critical indicators in evaluating a molecule's electronic structure, chemical reactivity, and photophysical properties [42]. The LUMO, which functions as an electron acceptor, is directly associated with the compound's electron affinity, whereas the HOMO, acting as the primary source of electron donation, correlates with its ionization potential. These orbitals together offer a comprehensive framework for understanding and forecasting the behavior of molecules under various environmental or reactive conditions. Using DFT, we calculated the spatial distribution and energy profiles of this FMO for **1** in the gas phase, as illustrated in Figure 8. The negative values of both HOMO and LUMO energy levels confirm the stability of complex **1** [33]. Notably, the FMO gap is calculated to be 1.39 eV for **1**. The HOMO–LUMO energy gap of 1.39 eV, although relatively low, falls within the range reported for related copper complexes, including 1.76 eV for mononuclear and 0.82 eV for binuclear Cu systems [43]. The reduced gap suggests significant electron delocalization and facile charge–transfer processes within the binuclear framework. Such behavior may be associated with enhanced electronic conductivity, increased chemical reactivity, biological activity, and potential photocatalytic activity. This low energy gap suggests a heightened electronic responsiveness, as electrons require minimal energy to transition between these orbitals [44]. The presence of two copper centers in **1** plays a critical role in modulating this electronic structure, markedly enhancing its chemical reactivity. This dual‐metal configuration not only facilitates greater orbital interaction but also promotes more efficient electron delocalization, which is a key factor in the compound's reactivity profile.

#### 3.5.4 Quantum Reactivity Analysis

Quantum chemical descriptors of the complex **1** was examined using DFT. This computational approach enabled the precise evaluation of several fundamental descriptors, including absolute hardness (*η *= (*E*_LUMO_−*E*_HOMO_)/2), absolute softness (*σ* = 1/*η*), global electrophilicity (*ω* = *μ* ^2^/2*η*), chemical potential (*μ *= −*χ*), additional electronic charge (Δ*N*_max_ = −*μ*/*η*), and absolute electronegativity (*χ *= −(*E*_HOMO_ + *E*_LUMO_)/2) [45]. These parameters collectively offer a comprehensive profile of the electronic nature and reactive tendencies of the complex. In particular, the interplay between hardness and softness elucidates the compounds’ resistance to charge transfer, while electrophilicity provides a direct measure of their tendency to accept electrons. The accurate representation of these properties underscores the power of DFT as a predictive tool for assessing chemical stability and reactivity in transition metal complexes, paving the way for rational design in coordination chemistry. The values of quantum reactivity parameters for **1** are estimated based on the following equations and summarized in Table 2.

**TABLE 2:** Quantum reactivity parameters of **1**.

Parameter	**1**
*E*_HOMO_	−5.31
*E*_LUMO_	−3.92
Δ*E*_(LUMO−HOMO)_	1.39
*χ*	4.61
*η*	0.69
*σ*	1.45
*μ*	−4.61
*ω*	15.40
Δ*N*_max_	6.68

The quantum reactivity parameters derived from DFT calculations reveal marked differences in the electronic behavior of the binuclear copper complex **1**. Complex **1** exhibits a significantly small HOMO–LUMO energy gap (Δ*E* = 1.39 eV), indicating a high chemical reactivity and low kinetic stability for **1**. This feature is further supported by the low absolute hardness (*η* = 0.69 eV) and high softness (*σ* = 1.45 eV^−1^), suggesting that **1** is readily undergoes charge transfer and responds dynamically to external perturbations. The global electrophilicity index (*ω*) of **1** (15.40 eV) is also markedly high implying a stronger electron‐accepting capability and great stabilization energy upon acquiring additional electron density. Additionally, complex **1** shows a high maximum charge transfer (Δ*N*_max_ = 6.68) reinforcing its superior electron‐accepting potential. The relatively high Δ*N*_max_ (6.68) and electrophilicity index *ω* (15.40 eV) are consistent with reported values for binuclear Cu(II) complexes (e.g., Δ*N*_max_ = 9.95 and *ω* = 20.30 eV) [43], supporting the strong electron‐accepting character of the present complex. Interestingly, the electronegativity (*χ*) values demonstrate high electronegativity (*χ* = 4.61 eV) for **1**, suggesting a strong tendency to attract electrons. Collectively, these descriptors confirm that **1** is chemically reactive and electrophilic, a finding that may be attributed to its binuclear architecture and the cooperative electronic effects between the two copper centers. These insights underline the utility of DFT in predicting and rationalizing reactivity trends in coordination complexes.

### 3.6 RDG/RDG Scatter

**FIGURE 9:**
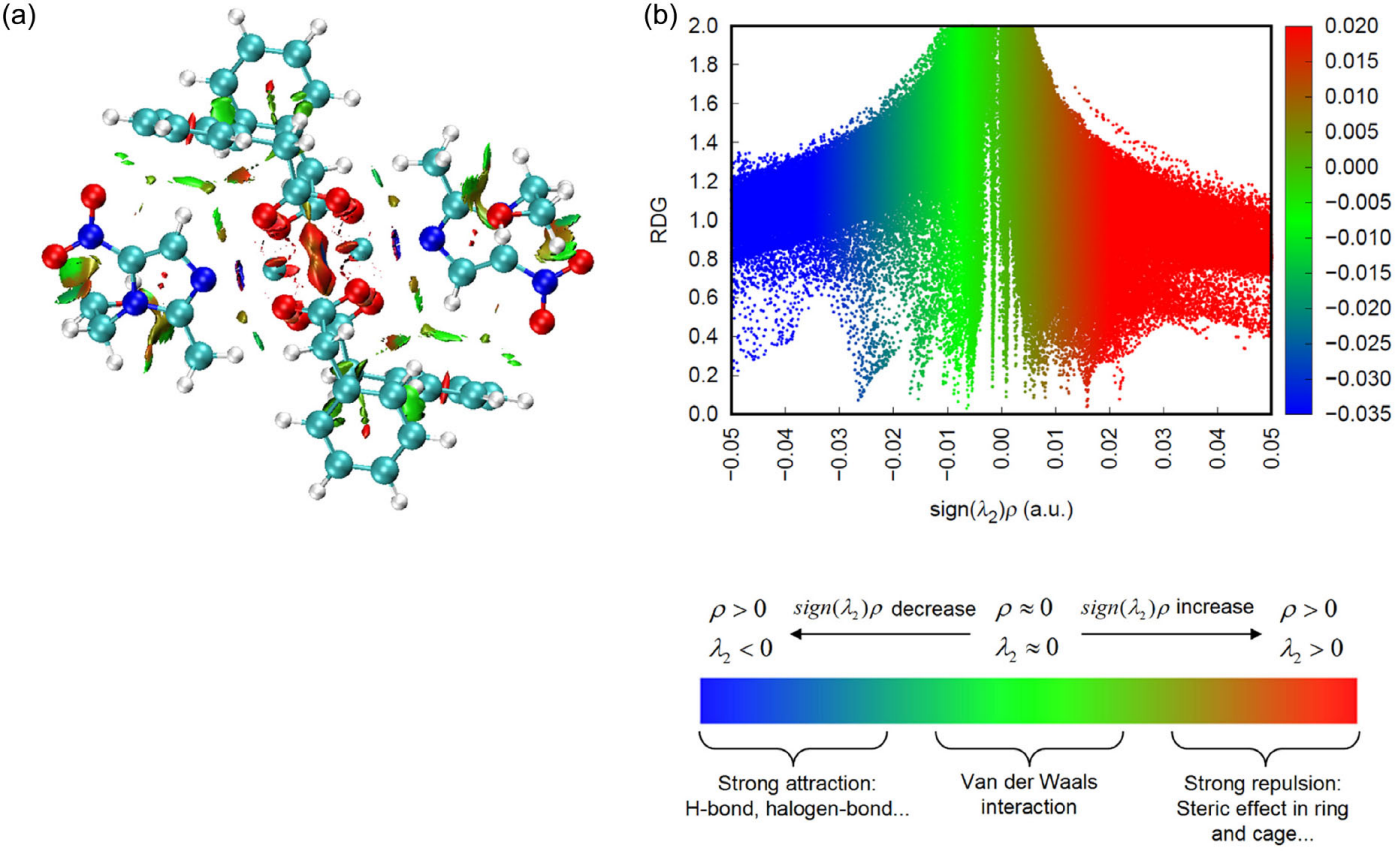
RDG (a) and RDG scatter (b) of copper(II) complex.

The noncovalent interaction characteristics of the metal complex were examined using RDG analysis together with the corresponding RDG scatter plot (Figure 9). The RDG isosurface reveals several distinct interaction regions distributed throughout the molecular framework. A pronounced red‐colored region observed between the central O···O atoms indicates strong steric repulsion arising from close spatial proximity of electron‐rich oxygen centers. Similar red patches appearing over the aromatic rings are attributed to intraring steric congestion and *π*‐electron density overlap, reflecting destabilizing interactions within the crowded molecular environment. In contrast, blue‐colored regions located between the nitrogen donor atoms and the copper metal center suggest attractive interactions associated with metal–ligand coordination, confirming the presence of stabilizing bonding interactions in the coordination sphere. These attractive features are consistent with negative values of sign (*λ*_2_)*ρ* in the RDG scatter plot, characteristic of electron density accumulation between interacting atoms. Additionally, extensive green isosurfaces are distributed across the structure, indicating weak dispersive interactions that dominate large portions of the complex. These regions correspond to vdW contacts and weak intermolecular or intramolecular stabilization effects, as supported by the near‐zero sign (*λ*_2_)*ρ* values in the scatter diagram. As a result, the combined RDG isosurface and scatter plot analyses demonstrate that the stability of the metal complex arises from a balance of coordination‐driven attractive interactions, weak dispersive forces, and localized steric repulsions.

### 3.7 ELF and LOL

**FIGURE 10:**
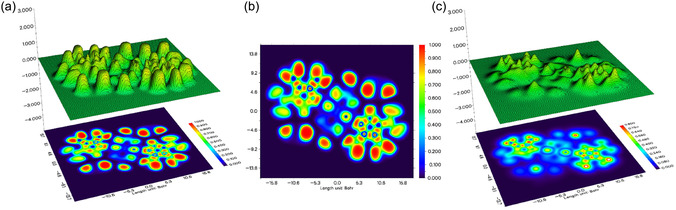
ELF (a,b) and LOL (c) of copper complex.

To obtain insight into the electronic structure and bonding characteristics of the copper complex, ELF and LOL analyses were performed (Figure 10). ELF values range between 0 and 1, where larger values indicate highly localized electron pairs, while smaller values correspond to regions of electron depletion or delocalization. Such analysis allows direct identification of bonding domains and nonbonding electron regions in coordination compounds [46]. As illustrated in Figure 10a,b, the ELF surfaces display dominant high‐value regions (red color) primarily surrounding hydrogen atoms rather than at the molecular core. Such localization is characteristic of *σ*‐bond electron density associated with C–H and N–H bonds, indicating strongly localized electron pairs along ligand frameworks. Additionally, moderate ELF domains distributed across the aromatic fragments reflect localized *π*‐electron regions consistent with preserved conjugation within the ligand backbone. The continuity of these regions indicates that coordination to copper does not significantly disrupt the intrinsic electronic structure of the aromatic rings.

The LOL map (Figure 10c) provides additional insight into orbital localization behavior. Notably, the region surrounding the copper center appears predominantly blue, corresponding to low LOL values and therefore reduced electron localization. This feature indicates that localized orbitals are not centered on the metal atom, implying limited accumulation of localized electron density in the vicinity of copper. In contrast, distinct localization regions are observed throughout the ligand framework, particularly along bonding regions and donor atoms, demonstrating that orbital localization is primarily ligand‐based. The absence of high LOL values near the copper center supports a bonding description dominated by ligand‐to‐metal donation rather than strong covalent electron sharing. Such behavior is characteristic of transition‐metal coordination complexes in which metal–ligand interactions arise mainly from polarized donor–acceptor interactions involving ligand lone pairs and metal orbitals. As a result, the combined ELF and LOL analyses indicate that the stability of the complex originates from localized ligand electron density coupled with directional coordination interactions.

Furthermore, no pronounced ELF maxima were observed directly along the Cu–O or Cu–N bond paths, suggesting that these interactions possess limited shared‐electron (covalent) character and are better described as predominantly polarized donor–acceptor coordination bonds. This observation is consistent with the low LOL values around the copper center. The reduced orbital localization surrounding Cu(II) can also be associated with the Jahn–Teller distortion expected for the d^9^ electronic configuration, where axial/equatorial bond asymmetry promotes uneven electron distribution and decreases localization at the metal center. Thus, the ELF/LOL results further support that metal–ligand bonding in the complex is influenced by both donor coordination interactions and Jahn–Teller‐induced electronic distortion.

### 3.8 Molecular Docking of **1** Against SARS‐CoV‐2 Main Protease (Mpro)

**FIGURE 11:**
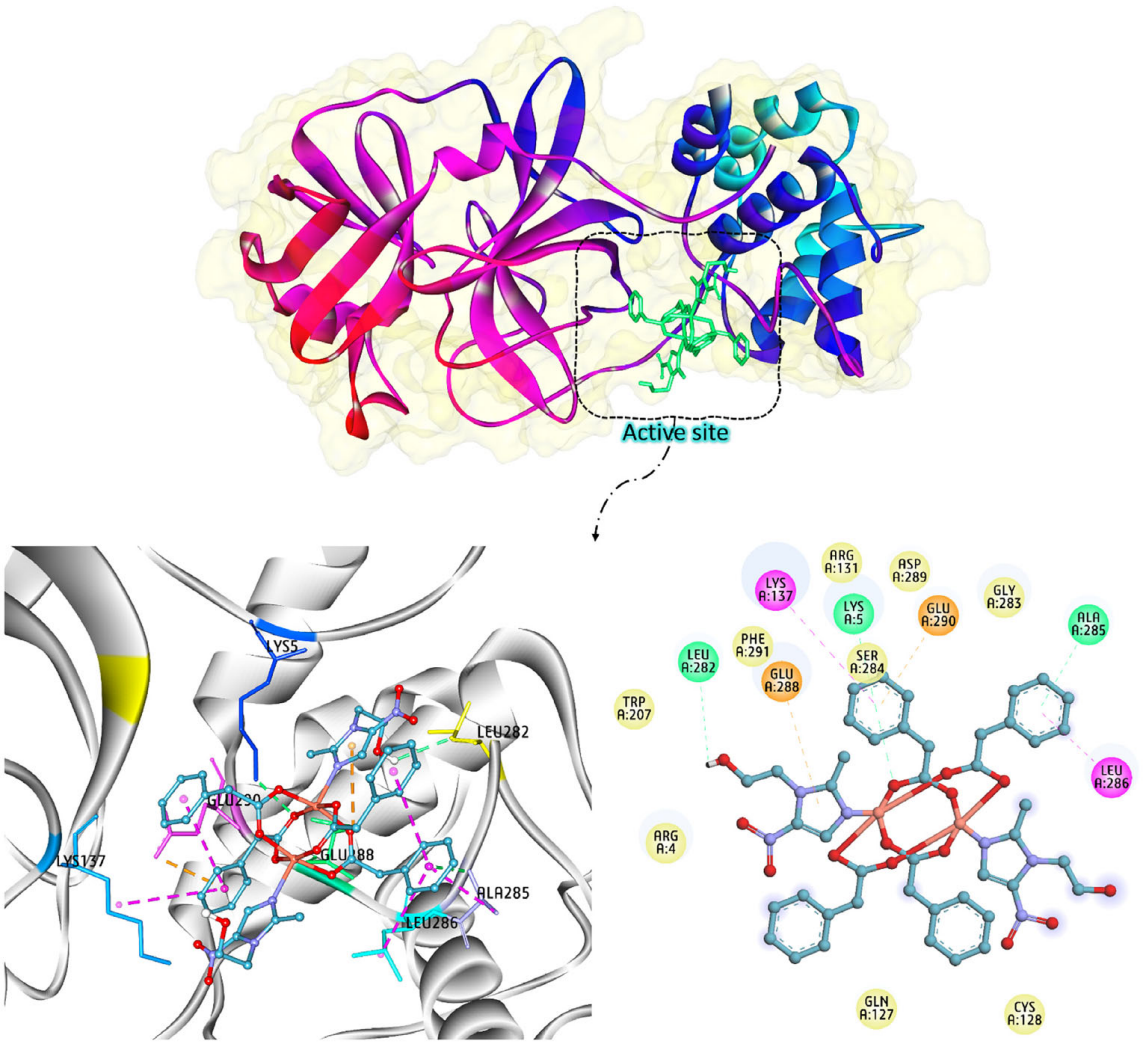
Docking perspective of binuclear copper complex.

To explore the antiviral potential of novel copper‐based complex **1** versus SARS‐CoV‐2, a molecular docking study was conducted, leveraging the established therapeutic relevance of copper coordination compounds. The crystal structure of the SARS‐CoV‐2 main protease (Mpro, PDB ID: 7BQY) was obtained from the Protein Data Bank and selected as the molecular target because of its essential role in viral replication and its importance as a therapeutic target. Computational docking simulation was done to assess the binding affinity and interaction profile of **1** with the 7BQY protein. The docking results revealed that complex **1** effectively interacts with the protein's active site, displaying notable binding free energy of −7.97 kcal/mol. This finding suggests that the Cu(II) complex may inhibit the catalytic function of SARS‐CoV‐2 main protease and thereby interfere with viral replication. Detailed structural analyses indicate that the complex establishes key interactions with amino acid residues associated with protease binding and inhibition, supporting their candidacy as potential inhibitors. The spatial interaction networks of the complex within the protein active site are illustrated through 2D and 3D representations (Figure 11), revealing further insights into possible protease inhibition mechanisms. Molecular docking analysis reveals that the interactions between compounds **1** and the 7BQY receptor are governed by a complex network of noncovalent forces, including hydrogen bonding, hydrophobic effects, vdW interactions, and electrostatic attractions. Compound **1** demonstrates a multifaceted binding profile. It establishes hydrogen bonds primarily with residues Leu282, Lys5, and Ala285, facilitating stable anchoring within the receptor's active site. Additionally, hydrophobic contacts are observed with Leu286 and Lys137, which contribute to the compound's structural accommodation. Electrostatic attractions are notably formed with residues Glu288 and Glu290, enhancing binding affinity. A broad range of vdW interactions further supports **1**'s conformational compatibility, involving Arg4, Trp207, Gln127, Cys128, Phe291, Ser284, Arg131, Asp289, and Gly283. These findings underscore the nuanced and multifactorial nature of ligand‐receptor binding for the title complex **1**, suggesting valuable insights into its potential as candidate SARS‐CoV‐2 main protease inhibitor. Such detailed interaction profiles provide a foundation for future structure‐based drug design and optimization efforts.

## 4 Conclusions

A novel copper(II) complex **1** was synthesized and characterized using X‐ray diffraction, spectroscopy, and DFT calculations. Complex **1** exhibits a binuclear paddle‐wheel structure with square‐pyramidal geometry, hydrogen bonding, *π*···*π* stacking, and C–H···*π*/O–H···*π* interactions stabilize the supramolecular frameworks. HS analysis confirmed the dominance of H···H contacts and demonstrated contributions from hydrogen bonds and *π*‐stacking interactions. The combined crystallographic and HS analyses provide a coherent structure–property relationship that highlights the dominant role of weak intermolecular interactions in stabilizing these Cu(II) assemblies. FTIR and UV–Vis spectrum corroborated the ligand coordination modes and *d*‐*d* transitions. The combined DFT and interaction analyses provide clear insight into the structural stability and electronic behavior of the binuclear copper complex. Computational results confirm good agreement with experimental geometry and reveal enhanced electronic delocalization arising from the dual copper centers. The small HOMO–LUMO energy gap, low hardness, and high electrophilicity indicate significant chemical reactivity and efficient charge–transfer capability. RDG, ELF, and LOL analyses demonstrate that the stability of the complex originates from cooperative metal–ligand coordination, weak dispersive interactions, and ligand‐centered electron localization. Molecular docking studies further show favorable binding interactions with the SARS‐CoV‐2 spike protein, demonstrating the potential of the complex as a promising platform for future metal‐based bioactive compound design. Beyond establishing the structural and electronic features of this binuclear Cu(II) complex, these findings provide a useful platform for the rational design of related multifunctional metal‐based systems with tunable photophysical and biological properties. In particular, the combined experimental–computational approach presented here may guide future development of copper coordination compounds for applications in bioinorganic chemistry and antiviral drug discovery. Future work will focus on expanding ligand modifications to tune electronic behavior, validating the predicted biological activity through experimental assays, and exploring structure–activity relationships to optimize functional performance.

## Author Contributions

**Abiodun Atoyebi Ajibola**: conceptualization, investigation, methodology, formal analysis, writing – original draft, writing – review and editing. **Mehran Feizi‐Dehnayebi**: writing – review and editing, writing – original draft, visualization, validation, software, data curation. **Senem Akkoc**: validation, software, writing – review and editing. **Lesław Sieroń**: software, investigation, formal analysis. **Waldemar Maniukiewicz**: software, investigation, formal analysis, writing – original draft, writing – review and editing.

## Funding

The authors have nothing to report.

## Conflicts of Interest

The authors declare no conflicts of interest.

## Supporting information

Supplementary Material

## Data Availability

The data that support the findings of this study are available on request from the corresponding author.
