# Supplementary material for: Synthesis, Crystal Structure, Hirshfeld Surface, RDG, ELF, LOL, DFT, and Molecular Docking Studies of a Binuclear Copper(II) Carboxylate Complex
Source: ChemistryOpen. 2026 May 20;15(6):e70231. doi: 10.1002/open.70231 (PMC13239497; doi:10.1002/open.70231)
Supplement: Supplementary file 1 — Supplementary Material [file OPEN-15-e70231-s001.pdf]

Electronic Supporting Information

Synthesis, Crystal Structure, and Hirshfeld Surface, RDG, ELF, LOL, DFT, and Molecular Docking Studies of a Binuclear Copper(II) Carboxylate Complex

Abiodun Atoyebi Ajibola[a,*], Mehran Feizi-Dehnayebi[b*], Senem Akkoc[b, c*], Lesław Sieroń[d] and Waldemar Maniukiewicz[d,*]

[a]Department of Chemical Sciences, Thomas Adewumi University, P.M.B. 1050, Oko-Irese, Kwara State, Nigeria.

[b]Department of Basic Pharmaceutical Sciences, Faculty of Pharmacy, Suleyman Demirel University, Isparta, Türkiye

[c]Faculty of Engineering and Natural Sciences, Bahcesehir University, Istanbul, Türkiye

[d]Institute of General and Ecological Chemistry, Lodz University of Technology, Zeromskiego 116, 90–924 Lodz, Poland.

*Corresponding authors E-mail:
[a]abiodunaajibola@gmail.com; abiodun.ajibola@tau.edu.ng
[b] mehrandehnayebi@sdu.edu.tr
[b,c] senemakkoc@sdu.edu.tr
[d]waldemar.maniukiewicz@p.lodz.pl

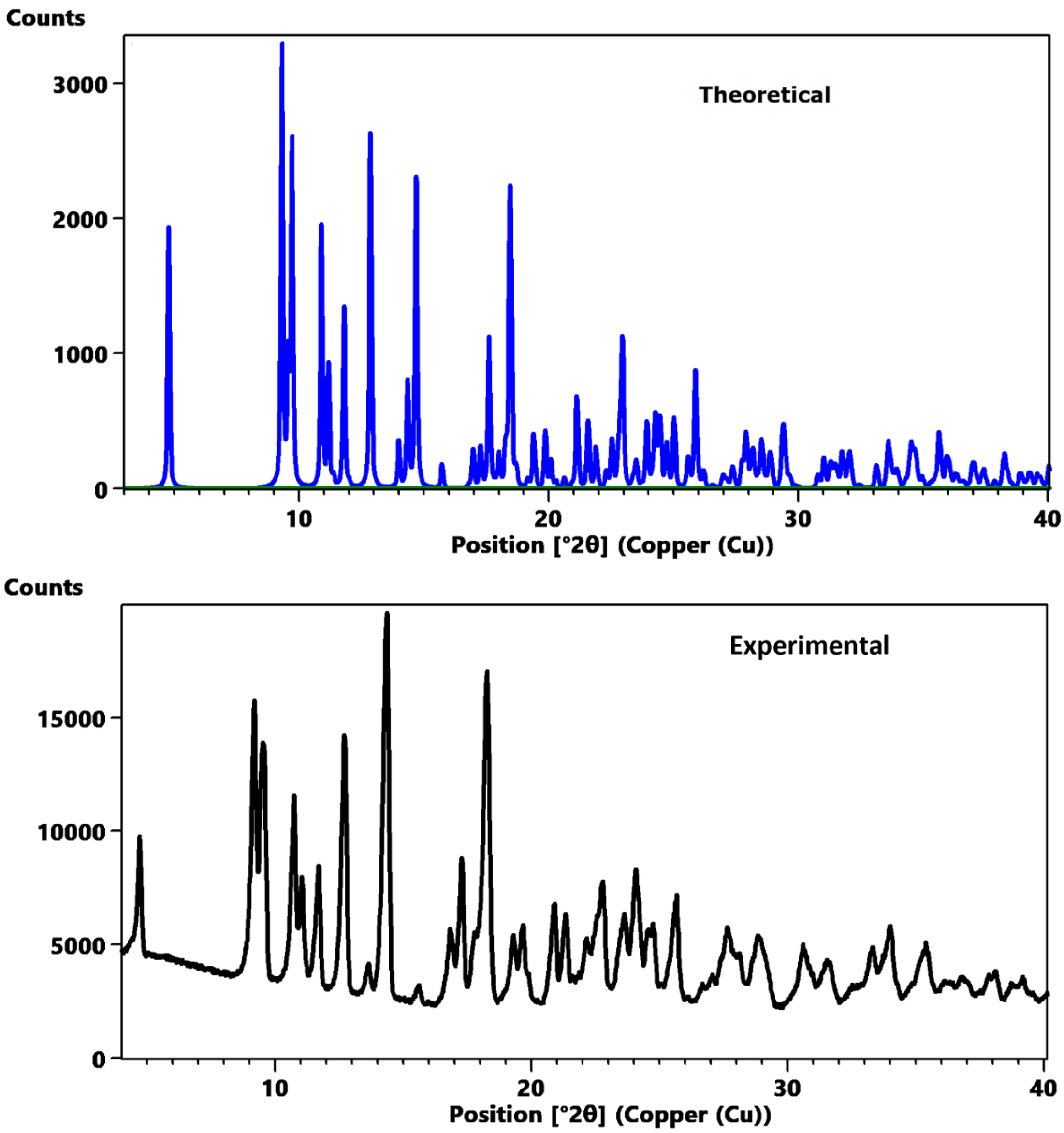

Fig. S1 Experimental and simulated powder XRD diffraction patterns of the complex **1**

**(1)**	**(2)**
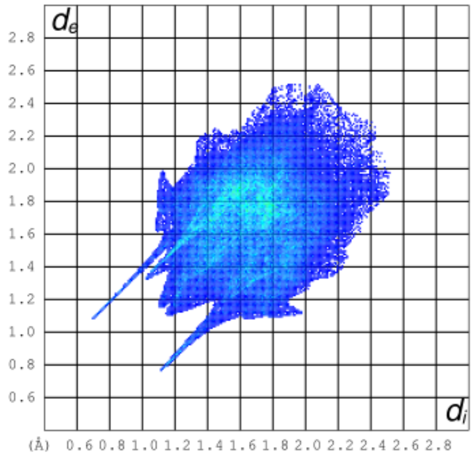 All	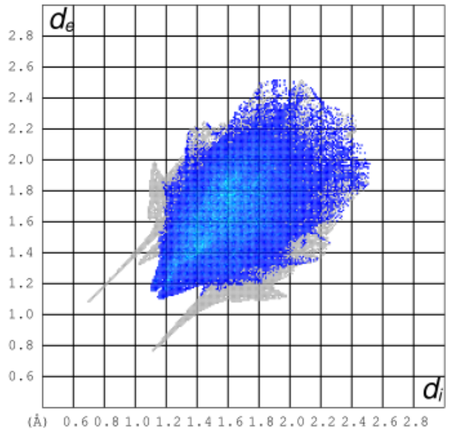 H···H 49.6%
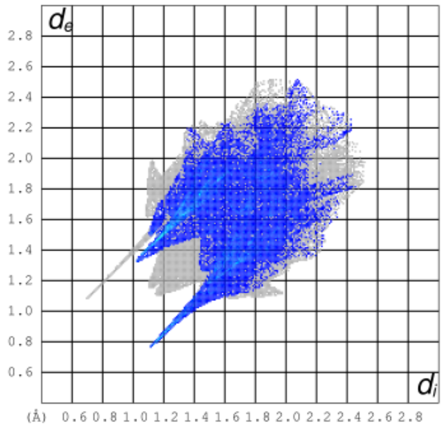 H···O/O···H 23.2%	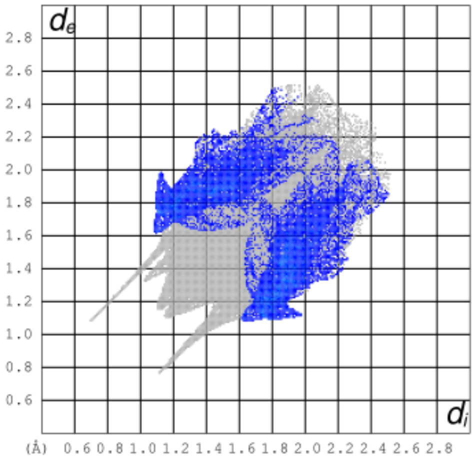 H···C/C···H 14.8%
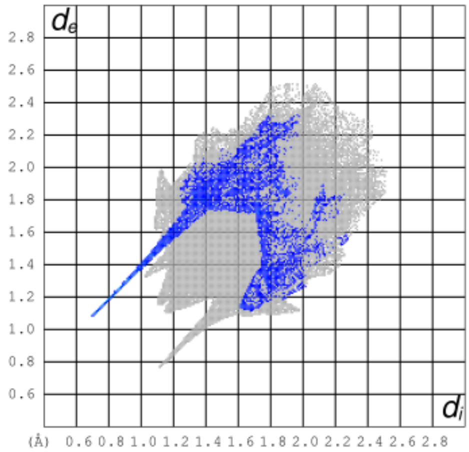 H···N/N···H 5.2%	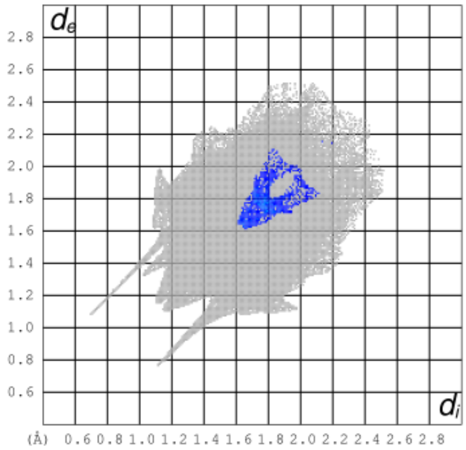 C···C 2.3%

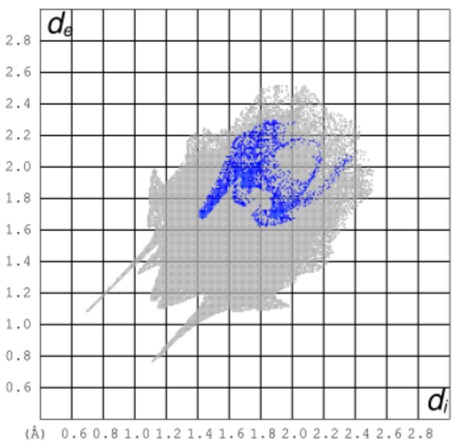

C···O/O···C 2.1%

Figure S2. The decompose 2-D fingers plot (>2%) for complex **1**

Table S1. Selected bond lengths (Å) and bond angles (º) for complex **1**.

**Atoms**	**1** **Bond Length (Å)**	**Atoms**	**1** **Bond Angle (º)**
**Cu1-O1**	1.9615(16)	O1-Cu1-O3	88.17(7)
**Cu1-O3**	1.9655(17)	O1-Cu1-N1	99.45(7)
**Cu1-N1**	2.1609(18)	O1-Cu1-O2$^{\#1}$	167.58(7)
**Cu1-O2$^{\#1}$**	1.9732(15)	O1-Cu1-O4$^{\#1}$	90.49(7)
**Cu1-O4$^{\#1}$**	1.9853(17)	O3-Cu1-N1	99.94(7)
		O2$^{\#1}$-Cu1-O3	91.26(7)
		O3-Cu1-O4$^{\#1}$	167.69(7)
		O2-$^{\#1}$-Cu1-N1	92.86(7)
		O4$^{\#1}$-Cu1-N1	92.35(7)
		O2$^{\#1}$-Cu1-O4$^{\#1}$	87.42(7)

Symmetry code: $^{(\#1)}$ 1-x,-y,1-z;

Table S2. Hydrogen bonding parameters for the complex **1**

**Compound**	**D-H···A**	**d(D-H)**	**d(H···A)**	**d(D···A)**	**<(DHA)**
**1**	O7-H7A···N4	0.74(4)	2.01(4)	2.750(3)	173(3)
	O10-H10···O11	0.80(3)	1.91(3)	2.695(2)	168(3)

	O11-H11A···O7^{I}	0.83(4)	2.02(3)	2.814(3)	160(3)
	O11-H11B···O4^{II}	0.81(3)	2.11(3)	2.903(2)	166(4)
	C20-H20B···O1	0.98	2.51	3.154(3)	143
	C21-H21B···O10^{III}	0.99	2.32	3.431(3)	155
	C22-H22B···O9^{IV}	0.99	2.42	3.240(3)	140
	C24-H24···O10^{I}	0.98	2.59	3.419(3)	146

Symmetry codes: $^{(I)}$ 1-x,1-y,-z; $^{(II)}$; x,1+y,-1+z; $^{(III)}$ -x,1-y,-z; $^{(IV)}$ -1+x,y,z

Table S3. π···π interactions for **1**

Compound	Interaction	Cg···Cg (Å)	Tilt angle α (°)	Slippage (Å)
**1**	Cg1···Cg3$^{\#1}$	3.659	4.75	1.5
	Cg4···Cg4$^{\#2}$	3.708	0.0	1.62

Symmetry codes: $^{\#1}$ -x,-y,1-z; $^{\#2}$ 1-x,1-y,-z.

1 - Cg1: N1-C18-C17-N2-C19; Cg3: C11-C12-C13-C14-C15-C16; Cg4: N4-C24-C23-N5-C25
Note: Cg···Cg is the centroid–centroid distance, α is the interplanar tilt angle, and slippage refers to the lateral offset between ring centroids. Interactions with Cg···Cg ≲ 3.9 Å and small α are considered significant π···π contacts.

Table S4. C–H···π and O-H···π interactions for **1**

Compound	Interaction	H···Cg (Å)	< X–H···Cg (°)	X···Cg (Å)
**1**	C20–H20C···Cg2$^{\#1}$	2.68	145.0	3.535
	O11–H11A···Cg1$^{\#2}$	2.95	113.0	3.843

Symmetry codes: $^{\#1}$ -x,1-y,1-z; $^{\#2}$ 1-x,1-y,-z

Cg1: N1-C18-C17-N2-C19; Cg2: C3-C4-C5-C6-C7-C8

Note: H···Cg is the distance from hydrogen to the aromatic centroid, X–H···Cg is the angle between donor–hydrogen–centroid, and X···Cg is the donor–centroid distance. Contacts with H···Cg < 3.0 Å and X–H···Cg > 110° are generally regarded as significant C–H···π interactions.
